# Scleritis Caused by *In Vitro* Linezolid-Resistant *Nocardia asteroides*

**DOI:** 10.1155/2014/326957

**Published:** 2014-10-27

**Authors:** Andres Gonzalez, Kaihan Fakhar, David Gubernick, Sonal Tuli

**Affiliations:** ^1^College of Medicine, University of Florida, 1600 SW Archer Road, Gainesville, FL 32610-0284, USA; ^2^Department of Ophthalmology, University of Florida, 1600 SW Archer Road, Gainesville, FL 32610-0284, USA

## Abstract

*Purpose*. To describe a case of postoperative scleritis caused by a novel strain of *Nocardia* resistant to linezolid and trimethoprim-sulfamethoxazole (TMP-SMX). *Methods*. Case report of a patient with microbiologically proven scleritis due to *Nocardia asteroides*. *Results*. The patient presented with pain, redness, and nodules on the sclera three months following pterygium excision with mitomycin C and amniotic membrane placement. As no response was noted with empiric treatment for bacterial scleritis, debridement was performed. The cytopathology report showed gram positive filamentous bacteria. A presumptive diagnosis of Nocardia scleritis was made and therapy was initiated based on a literature review on treatments for *Nocardia* infections. Cultures returned growing *Nocardia asteroides*. Antibiotic sensitivity testing revealed resistance to linezolid and TMP-SMX which are the traditional drugs of choice for *Nocardia*. The patient was treated with amikacin and imipenem as well as extensive debridement with pedicle grafts. The patient's scleritis resolved with a good visual outcome. *Conclusions*. Cultures should be obtained in all cases of necrotizing scleritis in patients with a recent history of conjunctival surgery to rule out unusual organisms such as *Nocardia*. Although literature states that resistance to linezolid and TMP-SMX is rare in *Nocardia*, sensitivity testing can be useful in unresponsive cases.

## 1. Introduction

The bacterial genus* Nocardia* includes a number of species which are Gram positive, aerobic, and filamentous shaped. Nocardia species typically only cause disease in immunocompromised individuals; however, a number of* Nocardia* infections have been reported in the immunocompetent including pulmonary nocardiosis and ocular infections such as keratitis and scleritis. The current first line antimicrobial treatment in cases of Nocardia scleritis includes amikacin and/or trimethoprim-sulfamethoxazole (TMP-SMX) [1]. In refractory* Nocardia* infection, linezolid is typically the antimicrobial agent of choice, as it has demonstrated near 100% sensitivity [2–4]. Our case represents the first report of Nocardia scleritis with* in vitro* resistance to linezolid as well as the second reported case of a Nocardia scleritis with* in vivo* resistance to TMP-SMX.

## 2. Case

A 46-year-old female with no pertinent past medical history presented to the urgent eye clinic with complaints of right eye mass with pain, redness, and swelling which had progressed over 3-4 weeks. She also complained of blurring, flashing lights, and color swirling in that eye. She had undergone pterygium surgery with mitomycin C and amniotic membrane graft placement of her right eye 3 months prior to presentation.

**Figure 1:**
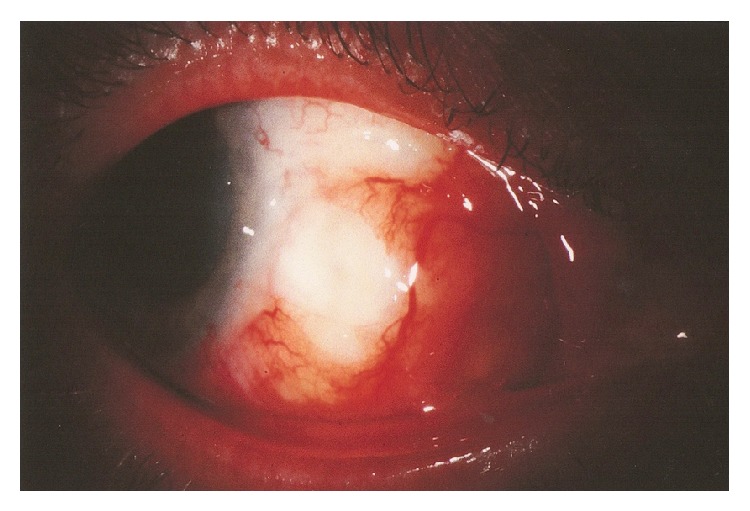
Slit lamp photograph showing area of redness and abscess formation on the temporal aspect of the globe.

On presentation, the patient was on an empirical treatment regimen of gatifloxacin ophthalmic drops four times a day (QID), prednisolone-acetate eye drops QID, and tobramycin-dexamethasone ophthalmic ointment QID. Initial examination showed inflamed, necrotic, and ischemic appearing sclera bordered by hyperemic and telangiectatic conjunctiva in the nasal quadrant (Figure 1). Uncorrected visual acuity was 20/20 vision bilaterally. Fundoscopic exam showed no frank inflammation but did reveal a slightly raised subchoroidal white region directly under the anterior segment lesion. A scleral biopsy and scrape of an inferior subconjunctival lesion was performed of the right eye and sent for microbiology and histopathology. One day later, pathology STAT called the ophthalmology department with gram stain showing gram positive beaded branching rods suspicious for* Nocardia*. Histopathology returned showing reactive conjunctival epithelium overlying the sclera with associated granulation tissue and focal tissue breakdown consistent with early abscess formation. A presumptive diagnosis of infectious nodular scleritis was made. Prednisolone-acetate eye drops were discontinued while gatifloxacin and tobramycin were continued. The patient was started on sulfacetamide 10% eye drops every two hours (q2h), polymyxin-trimethoprim eye drops QID, oral TMP-SMX twice daily (BID), oral doxycycline, and vitamin C for anticollagenase activity twice daily. Culture results returned negative four days after cornea scraping and biopsy was performed.

The patient continued to complain of increasing pain in the eye. Six days after initial presentation, she underwent scleral debridement due to expanding necrotic tissue that was presumed to be acting as a nidus for further infection. At this time, the vision in the right eye had declined to 20/40 −2. During surgery, it was noted that the entire inferior quadrant of the eye was ischemic and necrotic. A wide excision of the necrotic area was performed. Intravenous imipenem was given due to the presence of a very large area of exposed choroid. A pedicle graft consisting of conjunctiva and Tenon's capsule was used to cover the large area of exposed choroid. Imipenem 5 mg/mL drops were started every two hours. The patient continued to have significant pain and was taken back to the operating room one week later for further debridement as well as removal of a scleral abscess at the limbus. Cultures were drawn from this abscess during the procedure. Infectious disease was consulted and they recommended beginning amikacin due to anecdotal reports of efficacy in case series. Due to concern of invasive nocardiosis, parenteral imipenem was continued. Parenteral TMP-SMX was started to cover for possible resistance. Results returned five days later or three weeks after initial presentation with a diagnosis of* Nocardia asteroides*. Sensitivities of the* Nocardia* isolate showed resistance to TMP-SMX, sulfacetamide, clindamycin, and linezolid. It was sensitive to amikacin, azithromycin, and imipenem. All antibiotics were discontinued with the exception of imipenem IV every six hours (q6h), topical amikacin 2.5% q2h, and topical imipenem 5 mg/mL q2h.

**Figure 2:**
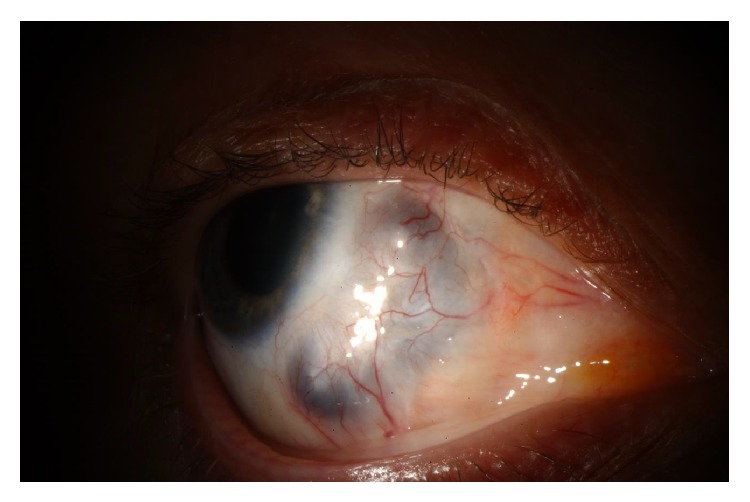
Slit lamp photograph of patient's right eye two months after initial presentation showing scleromalacia over initial locus of infection.

The patient reported improved vision and pain over the following 5 days and she was discharged with a peripherally inserted central catheter in order to continue her antibiotic regimen. Her medications were discontinued after eight weeks upon significant improvement. On resolution of the infection, the patient was left with an area of scleromalacia in the inferonasal quadrant where the sclera had been debrided (Figure 2). No further treatment was planned as the area was thought to be healthy and well-vascularized. Her uncorrected vision was 20/70. Safety glasses were advised at all times for protection of this potentially weak area.

## 3. Discussion

The predisposing factors for infectious scleritis are numerous. Literature has reported scleritis associated with sclera buckle surgery, trauma, cataract surgery, combined penetrating keratoplasty, and steroids [5]. Hodson et al. report that a history of pterygium excision along with concomitant radiation or mitomycin C is the most common predisposing factor for infectious scleritis in the study population [6]. The patient in this report developed infectious scleritis following pterygium excision likely due to ischemia from cauterization as well as nonhealing of the conjunctiva due to the concomitant use of Mitomycin C [7].

Our patient was initially treated with sulfacetamide eye drops, polymyxin-trimethoprim eye drops, and oral TMP-SMX based on recommendations from previous case reports. A frequently chosen option for ocular* Nocardia* infections is amikacin, likely due to its high susceptibility to the drug. Uhde et al. reported the resistance to amikacin to be as low as 5% [2]. Another treatment shown to be efficacious for nocardiosis is TMP-SMX, which has been specifically recommended in some reports [8–10]. When treating a case of refractory nocardiosis of the sclera, Decroos et al. endorse the use of linezolid due to the high susceptibility of* Nocardia* to the antimicrobial [11]. In fact, the literature reports that* Nocardia* has a near 100% susceptibility to linezolid with respect to all species and strains [2–4].

We report the first case of a Nocardia scleritis with a strain that is resistant to linezolid. Multiple studies have yielded only a handful of* Nocardia* isolates resistant to the antimicrobial [4]. This strain was also resistant to TMP-SMX. This resistance to TMP-SMX is the second reported case of a Nocardia scleritis in the literature [12].

We also report the first treatment of a Nocardia scleritis using dual therapy of topical amikacin and intravenous imipenem. Imipenem is not usually the first line therapy for* Nocardia* as this organism has been shown to be resistant to imipenem in up to 30% of isolates [2]. However, Ameen et al. reported that both imipenem monotherapy and imipenem in combination with amikacin on* Nocardia* infections refractory to sulfonamides were very efficacious; all patients in the study obtained at least a 75% improvement of their infection [13]. However, the use of this drug is limited by its cost, need for intravenous administration, and significant toxicity [3].

This case also underscores the need for debridement of infectious and ischemic tissue in cases of infectious scleritis—especially those caused by infectious organisms that are difficult to medically treat such as* Nocardia* or fungus. In addition, mobilizing vascularized tissue to the ischemic area by means of a pedicle graft can assist in resolving the infectious process.
